# Computational modeling of RNase, antisense ORF0 RNA, and intracellular compartmentation and their impact on the life cycle of the line retrotransposon

**DOI:** 10.1016/j.csbj.2021.10.003

**Published:** 2021-10-05

**Authors:** Michael David Martin, David N. Brown, Kenneth S. Ramos

**Affiliations:** a Dept. of Physics, University of Louisville, Louisville, KY 40214, United States; b Western Kentucky University, 1906 College Heights Blvd, Bowling Green, Kentucky 42101, United States; c Center for Genomic and Precision Medicine, Institute of Biosciences and Technology, Texas A&M Health Science Center, Houston, TX 77030, United States

**Keywords:** LINE-1, Retrotransposon, Reverse transcription, Stochastic modelling, Virtual Cell, Mass Action, LINE-1 ORF0, RNAi, Sequestration, Processing bodies

## Abstract

Nearly half of the human genome is occupied by repetitive sequences of ancient virus-like genetic elements. The largest class, comprising 17% of the genome, belong to the type 1 Long INterspersed Elements (LINE-1) and are the only class capable of autonomous propagation in the genome. When epigenetic silencing mechanisms of LINE-1 fail, the proteins encoded by LINE-1 engage in reverse transcription to make new copies of their own or other DNAs that are pasted back into the genome. To elucidate how LINE-1 is dysregulated as a result of carcinogen exposure, we developed a computational model of key elements in the LINE-1 lifecycle, namely, the role of cytosolic ribonuclease (RNase), RNA interference (RNAi) by the antisense ORF0 RNA, and sequestration of LINE-1 products into stress granules and multivesicular structures.

The model showed that when carcinogen exposure is represented as either a sudden increase in LINE-1 mRNA count, or as an increase in mRNA transcription rate, the retrotransposon copy number exhibits a distinct threshold behavior above which LINE-1 enters a positive feedback loop that allows the cDNA copy number to grow exponentially. We also found that most of the LINE-1 RNA was degraded via the RNAase pathway and that neither ORF0 RNAi, nor the sequestration of LINE-1 products into granules and multivesicular structures, played a significant role in regulating the retrotransposon’s life cycle. Several aspects of the prediction agree with experimental results and indicate that the model has significant potential to inform future experiments related to LINE-1 activation.

## Graphical abstract

**Figure:**
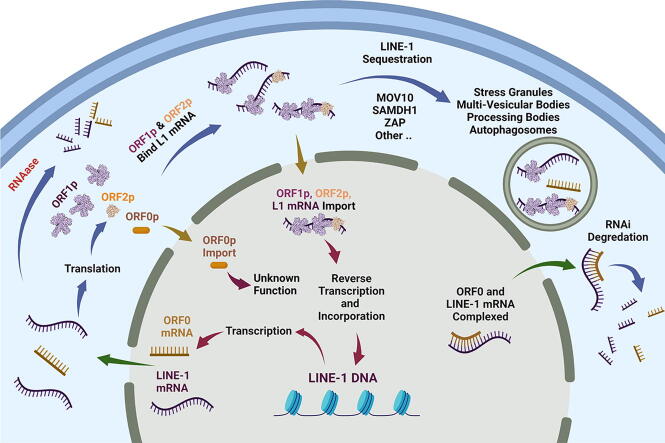

## 1 Introduction

There are an estimated 500,000 copies of the LINE-1 retrotransposon sequence in the human genome [1]; however, most are damaged with truncations, point mutations or other defects, leaving roughly 100 full-length LINE-1′s capable of transposition [2]. The length of human LINE-1 is ∼ 6kB and contains a 5′ untranslated region (UTR), two non-overlapping open reading frames labeled ORF1 and ORF2, followed by a 3′ untranslated region that ends with a polyadenylation signal [3]. The 5′ UTR has an RNA Pol II sense promoter and an antisense promoter [4] that was recently demonstrated to be translationally active, now dubbed ORF0 [5], [6]. ORF1 encodes for a 40 kDa RNA binding protein and ORF2 encodes for a 150 kDa protein (ORF2p) that has both endonuclease and reverse transcriptase activity [4], [7], [8]. The ORF2p has a cysteine rich region that binds to RNA in a non-specific manner [9], which participates in the process of reverse transcription. Mutations in either of these open reading frames have demonstrated that they are both necessary for functional retrotransposition [10].

**Fig. 1:**
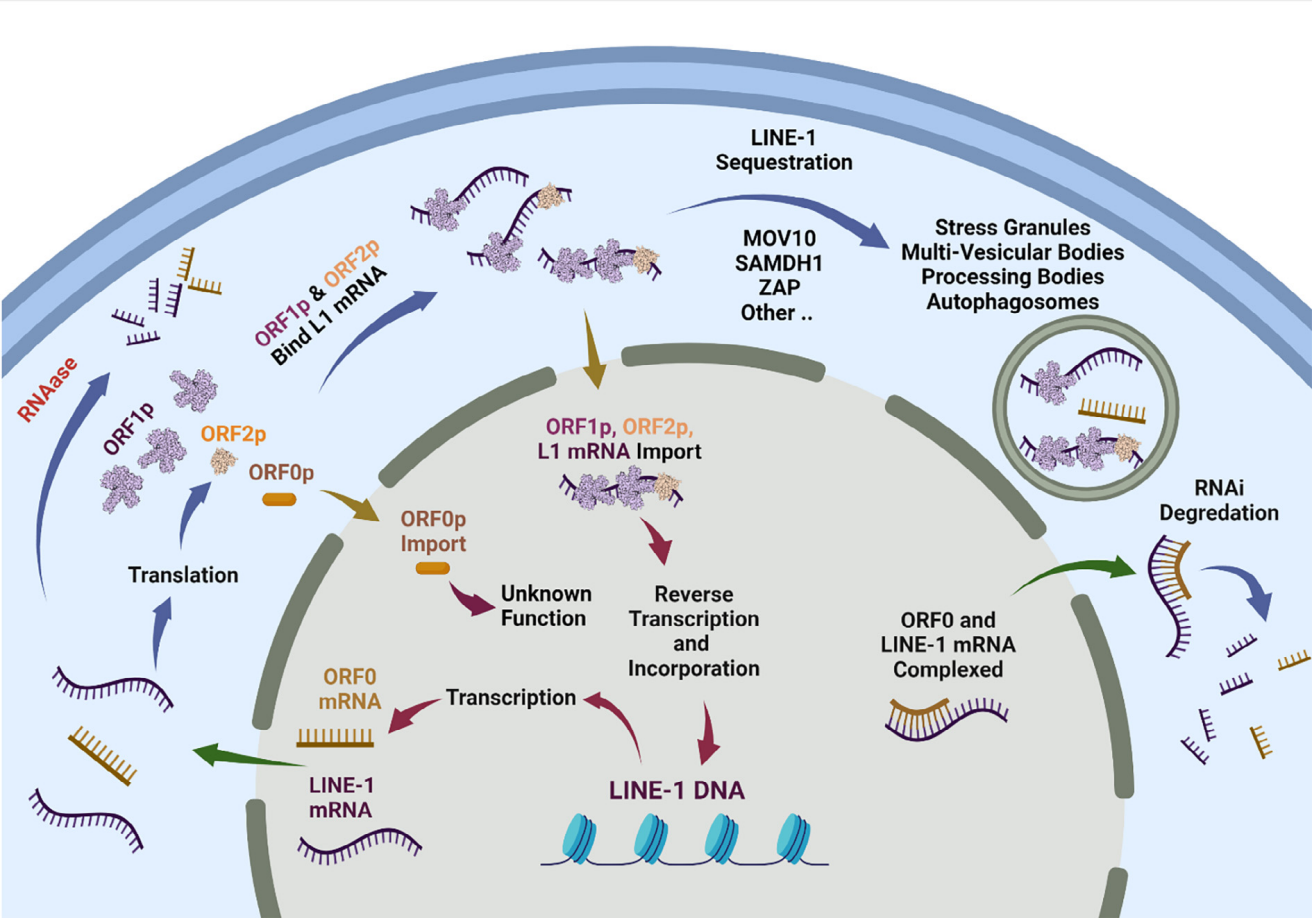
A simplified mechanism of the LINE-1 lifecycle and some cellular defense mechanisms.

As illustrated in Fig. 1, LINE-1 must be transcribed to RNA via RNA polymerase II, exported from the nucleus to the cytosol, then translated to generate the proteins ORF1 and ORF2 (ORF1p and ORF2p, respectively). These proteins then bind to their own RNA or to other RNAs, to form a ribonucleoprotein (RNP in Fig. 1). This complex is then imported back into the nucleus [11], where ORF1p acts as a nuclear chaperone, although the RNP complex may also access the DNA during cell division [12]. The ORF2p endonuclease domain then cleaves DNA at a degenerate consensus sequence (often rich in A/T nucleotides) [13], to leave a 3′ hydroxyl free for use as a primer. The reverse transcriptase domain of ORF2p synthesizes a cDNA that can be integrated back into the genome at the cut site [3].

The 5′ antisense promoter produces transcripts at a rate of about 1/8 that of the sense promoter [14] and yields dsRNA when binding to the complementary strand. Further, the ORF0 protein (only 71 amino acids long) has been suggested to play a role in LINE-1 mobility, and its overexpression shown to weakly increase in retrotransposon activity [5]. Antisense RNAs frequently serve many functions in cellular metabolism; in plasmids they can be used in copy number control and in phages they can inhibit primer formation [15]. Eukaryotes may use small complementary RNAs to manage splicing and in multicellular organisms antisense RNAs are widely used to manage embryonic development. They are known to influence chromatin organization and to control retrotransposon activity by blocking the activity of Reverse Transcriptase or RNase H [15].

LINE-1 is activated in the dysregulated genomes of cancer cells, although the exact relationship between cancer and retrotransposon activity has yet to be fully elucidated. Cell culture models of cancer have shown that pharmacologic inhibition of reverse transcriptase reduces proliferation of prostate, melanoma, teratocarcinoma, colon carcinoma, lung carcinoma, and acute myeloid leukemia cell lines in a dose dependent manner [13]. Tumor formation and progression may be linked to mechanisms related to LINE-1 metabolic activity. A carcinogenic insult can disrupt LINE-1 epigenetic silencing and lead to retrotransposition events that damage the genome or that more subtly influence tumorigenic progression [13]. For example, the 3′ UTR of LINE-1 mRNA interacts with a number of miRNA’s and may act either as a source or a sink for endogenous microRNAs that can dysregulate cellular metabolism [16], [17], [18], [19]. Finally, retrotransposon proteins, RNA and DNA are found in freely circulating exosomes (micro-vesicles) generated by tumor cells. Such exosomes may lead to lateral gene transfer and this is a potential cancer progression mechanism. Blood-borne exosomes are being actively investigated as an early stage cancer biomarker [19], [20], [21].

Given LINE-1′s propensity to disrupt genomes, the expression of these elements is regulated at several levels in tissue- and context-specific manners. At the epigenetic level, methylation of DNA CpG islands, histone acetylation and histone methylation are prominent. In the male germline, LINE-1 is inhibited by an elaborate system that includes PIWI-interacting RNAs (piRNA) leading to methylation of LINE-1 CpG sequences [13], [22], and related PIWI proteins for piRNA production. It is noteworthy that this secondary system of gene silencing is necessary in the germline, as typically DNA methylation is stripped during formation of primordial germ cells [23].

In addition to epigenetic mechanisms for regulating the transcription of LINE-1, cells have a host of other tactics for managing LINE-1 mRNA activity. In a recent review, over 100 proteins were described to interact with LINE-1 RNPs [12]; some of these are summarized in Table 1. As with other cellular RNAs, the action of RNases are principal degradation pathways for LINE-1 mRNA. In addition to the general degradation due to cellular RNase, RNase L (latent) is involved in an interferon-regulated pathway that responds to RNA and DNA viruses and has been shown to limit retrotransposition of LINE-1 in cell culture [24]. Similarly, RNase H2, localized to the nucleus, seems to play a role in LINE-1 RNA degradation of DNA:RNA duplexes [25], [26]. Innate cell defenses such as autophagy, the process of self-eating that utilizes special compartments known as lysosomes, are also implicated in the degradation of LINE-1 RNA. A different set of mechanisms are used to modulate the action of LINE-1 ribonucleoprotein particles (RNPs) that involve their localization in cytoplasmic stress granules [27]. These particles are associated with a cellular stress response where RNPs and mRNAs are degraded. A protein known as SAMHD1 and an RNA helicase, MOV10, potentially complexed with a zinc-finger antiviral protein ZAP, associate with the LINE-1 RNP localized in granules [3], [21]. A remaining open question is whether the localization of LINE-1 components in the cytoplasm is a necessary event in their “life cycle” as found in the yeast Ty3 retrotransposon [28] or a result of cellular defenses Table 2.

**Table 1:** Pathways used to manage epigenetic silencing and post-transcriptional components of LINE-1.

Pathway	Proteins	Compartment	Cell Type/Context	Description
RNase	RNase L, RNase H2, other cellular RNases	Cytoplasm, Nucleus	Demonstrated in human ovarian cancer cells, HeLa, HEK 293 T and SW982 cells	Cleavage of mRNAs from dsRNA [24] and RNA:DNA duplexes [25]
RNAi	Dicer, Exportin, RISC (contains Argonaute, others and siRNA)	Cytoplasm	All	Known to be generated by sense and antisense transcripts, as found in LINE-1. Plays a role in maintaining methylation H3k9me3 [29], [33], [41]
Granule Localization	MOV10, ZAP26, SAMHD1 [42]	Cytoplasm, stress granules and multi-vesicular bodies	All	General pathways involving localization of LINE-1 proteins, RNAs, and DNA in stress granules and multivesicular bodies [27]. Pathway involved in degradation, exosome formation and potentially processing bodies [42].
PIWI Interacting RNAs (piRNAs)	DNMT3L, PIWIL1, PIWIL2, PIWIL4 [22](aka murine MIWI2)	Nucleus, cytoplasm	Neoplasms, male germ line [13]. Demonstrated to play a role in HBEC LINE-1 propagation [37]	Leads to methylation of genomic LINE-1, members of the Argonaute family requires piRNA as a guide RNA.
apolipoprotein B mRNA editing enzyme [13]	APOBEC3A, APOBEC3B and APOBEC3C [42], [43], [44]	Nucleus	All, studies in HeLA	Inhibits reverse transcriptase (eg. Vif-deficient HIV-1 viruses are suppressed), Cytidine deaminase that converts dC to dU on forming DNA minus strands during reverse transcription.

**Table 2:** A list of typical initial conditions for the simulations. All other species are zero.

Species	Structure	Depiction	Clamped	Initial Condition
RISC_Cyt_Unbound	Cytoplasm		No	1000 [molecules]
MOV10_Zap_Cyt_Ubound	Cytoplasm		Yes	1000 [molecules]
L1mRNA	Nucleus		No	0 [molecules]
L1DNA	Nucleus		No	100 [molecules]
Exportin_Nuc_Unbound	Nucleus		No	1000 [molecules]
Dicer_Cyt_Unbound	Cytoplasm		No	1000 [molecules]

RNA interference (RNAi) mechanisms may play a role in post-transcriptional degradation of LINE-1 RNA [14], and its epigenetic silencing. RNAi targets double stranded RNA; the double strand may be formed by mRNA transcribed from the ORF0 antisense promoter [29] within the 5′ UTR of LINE-1 [14] binding to LINE-1 mRNA, although recent evidence suggests that overexpression of ORF0 leads to a modest increase in LINE-1 mobility [5], [6]. Double stranded RNA (without a hairpin in this case) is exported from the nucleus and targeted by the enzyme Dicer to form 22 nucleotide siRNAs [30], [31]. Then a helicase separates the two complementary strands of the siRNA, one of which is subsequently loaded into the RNA induced silencing complex (RISC) that includes the Argonaute and Slicer proteins. There is further evidence that RNAi related pathways are critical to the maintenance of heterochromatin (condensed chromatin) structure in genomic regions containing transposons and repetitive elements [32]. Currently, it is not clear what the relative roles of these two RNAi pathways are on LINE-1 management [33].

Finally, the cell may target the reverse-transcribed complementary DNA (cDNA) for degradation. The APOBEC3 enzyme catalyzes conversion of cytosine in DNA to uracil via deamination (and evidently oxidation) and may then enable degradation via endonuclease activity [34], [35], [36]. Further, the cDNA can be directly degraded via the endonucleases TREX1 and ERCC1/XPF [3].

Experimentally, the dynamics of LINE-1 activity have been explored by introducing the tobacco smoke carcinogen benzo(a)pyrene (BaP) to cells in culture and subsequently monitoring the time evolution of LINE-1 mRNA. In one study utilizing human bronchial epithelial cells (HBEC), the time course of LINE-1 activation was shown to peak at 12 h and return to base line by 48 h after exposure to BaP [37]. In another study, the time course of LINE-1 mRNA was quantified in HeLa cells after exposure to BaP [38]. The data showed that LINE-1 mRNA rises from 5.24 AU (untreated control) to a near constant level of ∼ 11 AU after 3 h and remains roughly constant until the end of the experiment at 96 h. These experiments are particularly relevant to the present study as we explore the dynamics of LINE-1 as a function of both the rate of LINE-1 mRNA creation and as a function of initial LINE-1 mRNA. These parameter sweeps are intended to model the influence of carcinogen mediated epigenetic dysregulation on the fates and dispositions of LINE-1 components.

## 2 Previous simulations

Previous simulations of LINE-1 activation by Rempala et al. [38], [39] focused on the steady state solutions of relatively simple systems. These included reactions involving the creation of LINE-1 mRNA from the corresponding DNA, the formation of a single protein from the mRNA and the creation of complementary DNA. While both continuous and stochastic models were explored, the highly simplified architecture of the model was a shortcoming of the work. Other explorations of the RNAi machinery have demonstrated that complex behavior emerges due to feed forward and backward loops between miRNA and their targets, even for relatively simple systems, thus making computational models particularly critical for exploring these complexities [40].

## 3 Goals of the present simulation

A principal goal for the present study was to examine the relative roles of various novel mechanisms for LINE-1 post-transcriptional regulatory control, particularly within the context of cancer cells where LINE-1 is dysregulated. A model was designed to gain additional insight into the dynamics of LINE-1 components such as mRNA, ORF0 mRNA, and ORF1 and ORF2 proteins. The simulation specifically explored how the positive feedback loop inherent to LINE-1 interacts with general cytoplasmic RNase activity, RNA interference pathways, and processes involved in sequestration of LINE-1 into stress granules and multi-vesicular bodies associated with exosome formation. Such a model facilitates direct comparison to experiments where carcinogens such as BaP are introduced into cells in culture to monitor the time evolution of LINE-1 products. Acute exposure to the carcinogen has been demonstrated to effectively disrupt epigenetic silencing of LINE-1 resulting in transient expression [45], [46]. Finally, it was our intent to prepare an open model that provides a starting point for enumerating the regulatory mechanisms involved with LINE-1. It is important to note that the model does not include the role of epigenetic silencing, the activity of deaminases such as APOBEC, the piRNA pathway, or the role of the ORF0 protein in affecting transposition efficiency.

## 4 Model components and framework

Models were created within the free Virtual Cell package developed by the University of Connecticut [47] and available via the application from a shared model database. The modeling environment readily enables the creation of reaction networks that occur within user defined cellular compartments. The networks may be modeled using a variety of algorithms including continuous (differential equation based), stochastic, and network free approaches [48]. Further, the models may be constructed so that BioNetGen can be employed to automatically generate permutations of individual reactions that involve formation of complexes or a multitude of species [49], [50]. This allows, for example, formation of polymers such as actin, or accounting for cases where a large variety of proteins may be degraded by a single protease. Only models that are fully expressed as mass action kinetics are currently compatible with BioNetGen and the agent-based network-free algorithms. The Virtual Cell environment also enables integration of fully spatial models that can include full Monte Carlo representations.

In this work, only non-spatial models were used with transport between compartments occurring via a simple mass action reaction. Thus, the system is assumed to be well-mixed and does not incorporate diffusion of molecules. Given that the number of molecules, e.g., “Hot” LINE-1 DNA copies or LINE-1 mRNAs, may be < 100 at different time points, stochastic simulations were deemed to be more appropriate than continuous simulations. Solutions for stochastic problems were found using the Gibson-Bruck algorithm [51] that converts reaction constants to probabilities and is a refinement of Gillespie’s methodology [52]. However, due to limitations in stochastic modeling within Virtual Cell, the full system of reactions must be written in terms of elementary mass action, as opposed to more sophisticated reaction models such as Michaelis-Menten or cooperative Hill binding, though these may be converted to mass action expressions.

The present model was initially constructed from three separate components: a representation of the LINE-1 life cycle, RNA interference, and an approximation of pathways involving proteins responsible for cytosolic RNA sequestration into stress granules and pre-exosomal multi-vesicular bodies. Each system was computationally explored, and in the case of Dicer with the RNA-induced silencing complex (RISC) from the RNAi pathway, the kinetics matched to experimentally derived rates. The constants used in the models are provided as supplementary information.

## 5 RNA interference model

**Fig. 2:**
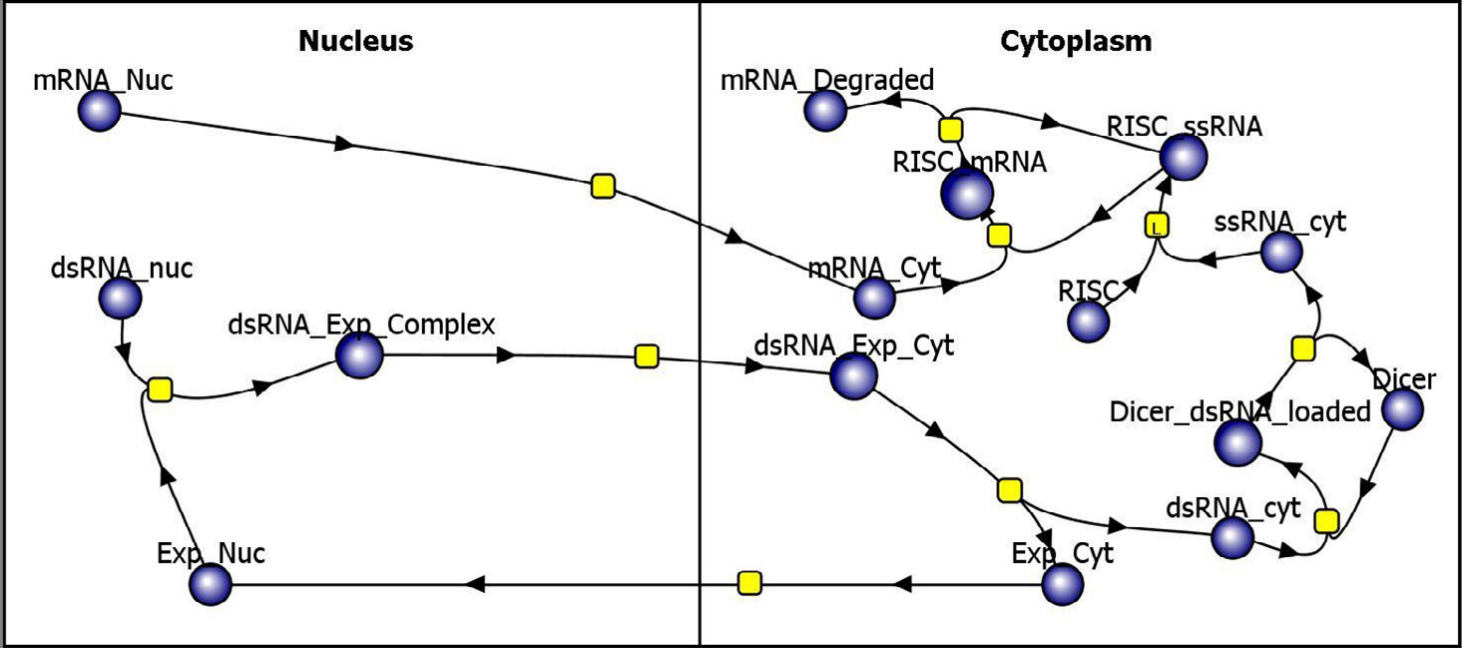
Illustration of the RNAi reaction pathway used to represent the degradation of perfectly complementary dsRNA generated by LINE-1 ORF0 RNA (or other fully complementary RNA) and LINE-1 mRNA. The blue circles are reactants and product species, the yellow squares are reactions and the arrows dictate the direction of the reaction. (For interpretation of the references to colour in this figure legend, the reader is referred to the web version of this article.)

Fig. 2 illustrates the RNA interference sub-model containing only seven unique molecules. The blue circles symbolize reaction species, and the yellow squares symbolize reactions. In the top left of the figure, mRNA (denoted as mRNA_Nuc) is exported from the nucleus via mass action (although a chaperone mechanism was also explored) to the cytosol where it may then be incorporated into RISC primed with a complementary ssRNA. In the figure, the complex formation is symbolized by the reaction of mRNA_Cyt with RISC_ssRNA to form RISC_mRNA. The values for the Michaelis-Menten RISC kinetics were taken from Haley et al [53] and are valid for a RISC complex with a fully complementary siRNA loaded, although it should be noted that there is evidence suggesting that the kinetics may be specific to the guide RNA. In the nucleus, dsRNA (dsRNA_nuc) in the center left of the figure, representing LINE-1 mRNA complexed with the antisense ORF0 mRNA, is ferried from the nucleus with the Exportin protein [29], [31] (dsRNA_Exp_Complex in Fig. 2). The dsRNA disassociates from Exportin after arriving in the cytoplasm, the Exportin reenters the nucleus, while the dsRNA (dsRNA_cyt) may get loaded into the Dicer enzyme. In the model, Dicer represents the human Dicer1 enzyme found in the cytosol which is an RNAase that cuts pre-miRNA and dsRNA to lengths of 20 to 25 nt leading to the formation of single stranded, small interfering RNA’s (siRNA’s). It is noteworthy that the rates of cleavage are 100x higher for pre-miRNA containing a hairpin structure [54] versus those formed out of perfectly complementary dsRNA. We chose to use kinetics associated with fully complementary dsRNA without a hairpin [54], as would be expected for LINE-1 mRNA complexed with its perfectly complimentary anti-sense Orf0 RNA. The output of Dicer, denoted ssRNA in Fig. 2, is loaded into the RISC enzyme which catalyzes the cleavage of target mRNA that is complementary to the guide siRNA [53]. There is some evidence that versions of this complex also incorporate into MOV10, a protein implicated in RNA sequestration. Note that the Drosha enzyme is not represented in the model as it is specialized to the initial step of the RNA interference pathway and specific for stem-loop structured RNA [see Genecard entry].

The Michaelis-Menten kinetics for Dicer and RISC were converted to elementary reactions with mass action kinetics to enable stochastic models in the Virtual Cell [55], [56]. That is, the reaction:

$$E+S{↔}_{k_{-1}}^{k_{1}}ES\to^{k_{2}}E+P$$

in which an enzyme, E, binds to a substrate, S, with forward and reverse binding constants k_1_ and k _-1_ forming the enzyme-substrate complex ES which then catalyzes the formation of some new product, P. The classic Michaelis-Menten kinetic equation for which experimental data are frequently available and which can be modeled only as a continuous ordinary differential equation in Virtual Cell is given by

$$\frac{d[P]}{\mathrm{dt}}=\frac{\left[S\right]∙V_{\max}}{\left[S\right]+K_{m}}$$

wherein [S] is the concentration of substrate and [P] is the product concentration.

The conversion to a math model for enzymes with mass action kinetics typically follows one of two approaches: the quasi-steady-state and the equilibrium approximation. The equilibrium approximation assumes that the substrate concentration is in instantaneous equilibrium with the enzyme-substrate complex; whereas the quasi-steady-state assumes the amount of enzyme-substrate complex is constant and that the amount of enzyme is much smaller than the substrate [57], [58]. Here, we choose the equilibrium approximation as the catalyzation to the product (P) in Eq. 1 with constant k_2_ is much slower than the k_1_ reaction; see sheet 1 titled “Model Constants” in the supplementary information for the reaction constants. From the elementary reactions in Eq. 1,

$$V_{\max}=k_{2}∙E_{t}$$

and

$$K_{m}=\frac{k_{-1}}{k_{1}}$$

Thus, for purposes of converting the model’s enzymatic reactions from Michaelis-Menten to mass action kinetics

$$k_{2}=\frac{V_{\max}}{E_{t}}$$

and

$$k_{-1}=k_{1}∙K_{m}$$

where we choose an arbitrary k_1_ and scale k_-1_ by K_m_, specifically k_1_ was set to 10 (µM*s)^-1^ for both Dicer and RISC. As shown in Eq. 6, only the ratio k_-1_/k_1_ have an impact on the kinetics of the reaction. This is further demonstrated in the tab labelled “Mass Action Dicer” in the supplemental spreadsheet where we simulate the reaction velocity curves for k_1_ = 10 (µM*s)^-1^ and 100 (µM*s)^-1^ using an appropriately scaled k_-1_; the data are identical.

Validation of the model against experimental data is given in the supplementary spreadsheet. In the sheet titled “Michaelis-Menten Dicer” we first validated a continuous Michaelis-Menten Virtual Cell model for the action of DICER alone against the experimental data found in Chakravarthy et al [54]. Then the model was converted to a stochastic-competent, mass action representation and verified. The data are shown in the sheet titled “Mass Action Dicer”. Similarly, a model was created for the RISC complex cleaving perfectly complimentary mRNA and compared to experimental data from Haley and Zamore in the sheets titled “RISC ssRNA Michaelis-Menten” and “RISC ssRNA Mass Action”. In each case, the simulated data matched the empirical literature curves.

## 6 Exosome and stress granule model

**Fig. 3:**
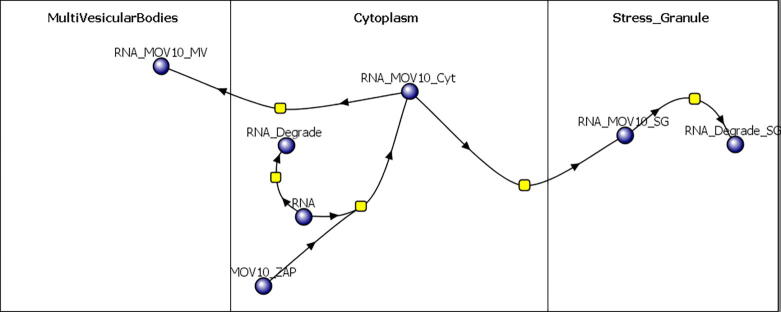
Basic model of stress granule and exosome formation related to exogenous RNA or retrotransposons.

This portion of the model is intended to capture a simplified pathway reflecting the dynamics of stress granule and exosome formation following LINE-1 activation. Fig. 3 illustrates the basic topology of the reaction network. In this model, the mediating localization protein is labeled “MOV10” and is used as a gross simplification of a multitude of pathways that may include ZAP, MOV10, or SMAHD1 . This is only a guess in as much as considerable information is yet to be learned about the details of localization in “processing bodies”, stress granules and exosomes [42]. The idealization, however, serves as a starting point for building future model topology in this emerging area of research.

As with the RNAi portion of the model, we first modeled the pathway independent of the larger LINE-1 model. In this model there are only 2 molecules: RNA and MOV10_ZAP and 3 compartments labeled: Cytoplasm, MultiVesicularBodies, and Stress_Granule. The RNA represents endogenous retrotransposon RNA (although many of these localization proteins also act on exogenous viral RNA^42^) and MOV10_ZAP is used as a simplified place-holder for the proteins involved in localization to the two compartments within the cytoplasm. The compartment labeled “MultiVesicularBodies” represents a large group of these precursors to exosome formation and the “Stress_Granule” represents structures that are involved in RNP sequestration and degradation. In the full model, this pathway is used for localization of LINE-1 ribonucleoprotein particles to these compartments.

The stand-alone sequestration model was run with 100,000 and 300,000 initial RNA molecules to explore the basic dynamics of the system. Graphs of the results and the raw data are presented as supplemental information in the spreadsheets labeled “Exosome Proc Bodies 100 k RNA” and “Exosome Proc Bodies 300 k RNA”. In both cases, only a small fraction of the RNA molecules end up in the “MultiVesicularBodies” or “Stress_Granule” compartments; only 2.4% of the RNA is sequestered into either of these compartments and 95.3% is degraded by background RNase activity in the cytoplasm.

## 7 LINE-1 life cycle model

**Fig. 4:**
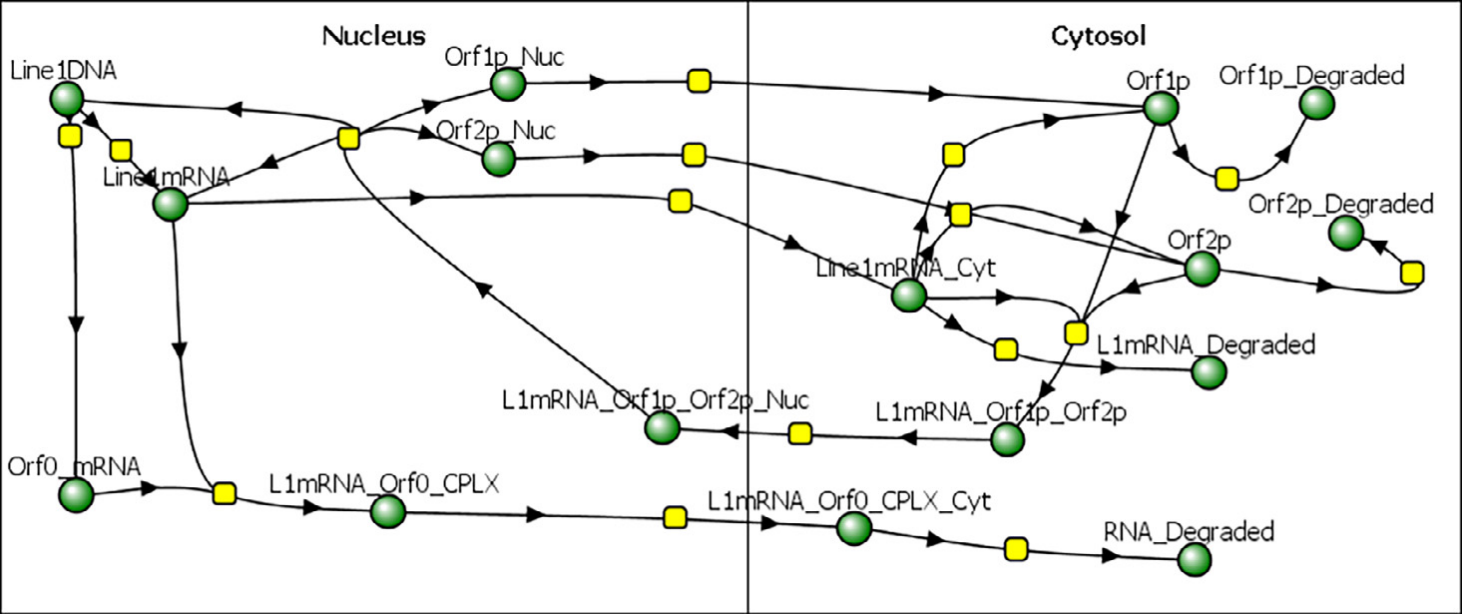
A simplified visualization of the LINE-1 life cycle model.

Fig. 4 illustrates a simplified model of the LINE-1 lifecycle used in this work. Starting in the top left of the diagram, upon dysregulation, “Line-1DNA” may generate “Line1_mRNA” but it may also generate the antisense “Orf0_mRNA”. Following ORF0 mRNA generation, it may complex with the complementary sense LINE-1 mRNA to form a double stranded complex, “L1mRNA_Orf0_CPLX”, that is subsequently exported from the nucleus and degraded by RNAi mechanisms in the cytosol (bottom of Fig. 4). Similarly, “Line1mRNA” may be degraded by general cytosolic RNase activity after nuclear export or it may be translated into the ORF1 and ORF2 proteins. These proteins subsequently bind to the LINE-1 mRNA, “Line1mRNA_Cyt”, to form a RNP that is imported back into the nucleus, “L1mRNA_Orf1p_Orf2p_Nuc” in the center bottom of the figure. In the model, LINE-1mRNA can only reenter the nucleus if it has at least 1 ORF2p and at least 1 ORF1p. The LINE-1 RNP can then reverse transcribe the LINE-1mRNA to produce a new copy of “Line1DNA”, essentially closing the life cycle of the genetic element. Afterwards, the ORF1 and ORF2 proteins are exported back to the cytoplasm to participate in another round of retrotransposition or be degraded; see top right of Fig. 4. Given that the ORF2 reverse transcriptase lacks RNase H activity [9], [59], the mRNA is preserved and may be re-exported to the cytosol where it may once again participate in RNP formation and production of new LINE-1 DNA. The assumption that the freshly reverse transcribed mRNA does not interact with ORF1p and ORF2p within the nucleus is based on the observation that neither protein tends to localize in the nucleus [27], [60]. The model only reflects the formation of new fully functional DNA copies of the LINE-1 element, when in reality many reverse transcribed copies are not complete.

Generally this model shows exponential growth of LINE-1 DNA copies as a function of time, in agreement with previous simulations [38].

## 8 Full model description

**Fig. 5:**
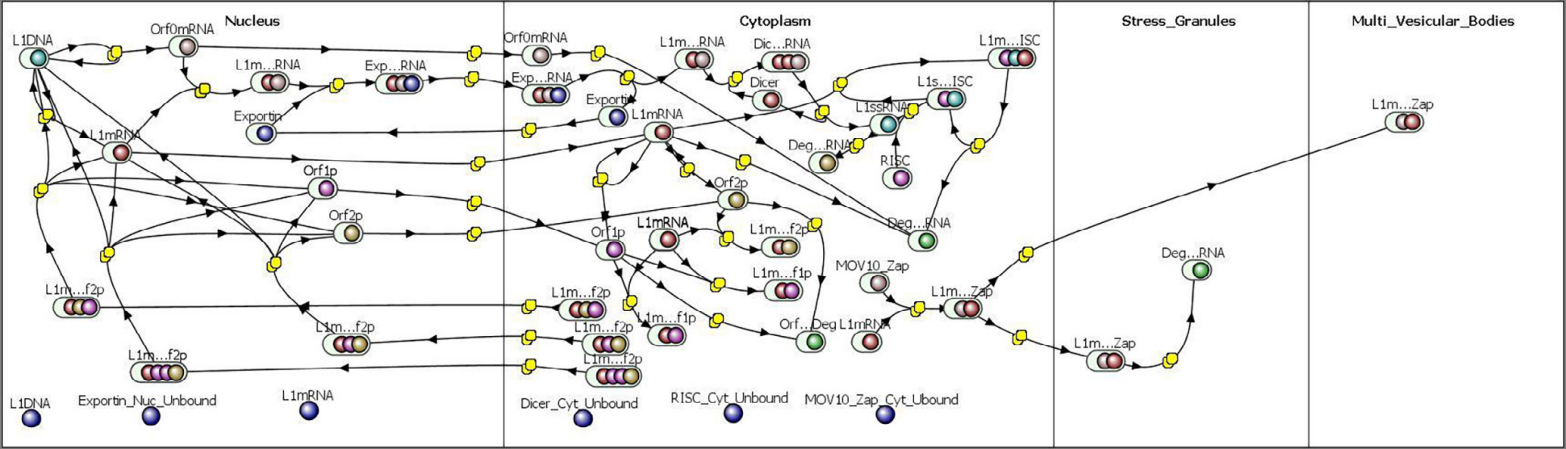
Reaction diagram of a fully integrated model. The ellipses with colored circles represent reactants and products that may be molecular complexes and have internal states. As before, the yellow squares represent reactions and the blue circles at the bottom of the diagram are species definitions used for setting initial conditions. A higher resolution image is provided in the supplemental information. Fig. 1S. (For interpretation of the references to colour in this figure legend, the reader is referred to the web version of this article.)

Each of the three submodels were combined using a mass action kinetic approach to maintain compatibility of stochastic simulations within the Virtual Cell environment. Fig. 5 illustrates the reaction network for the merged model. The yellow squares in the figure symbolize reaction rules and the ellipses with colored circles represent molecular species. The blue circles at the bottom of the figure are species in the model that are used to specify initial conditions for purposes of BioNetGen and Network Free simulation. The model contains a total of 13 different molecule types and 36 reaction rules, shown in Fig. 2S and Table 1S, respectively. In the full stochastic competent model, the molecules have specific binding sites that account for the formation of protein complexes. Of particular import is the LINE-1 mRNA that, as a simplifying approximation, contains only 4 binding sites: 2 for ORF1p, one for ORF2p, and a site for ORF0 mRNA binding. The approximation that a maximum of only 2 Orf1 proteins bind to a LINE-1 mRNA is made here to simplify the potential permutations of LINE-1 mRNA states. Experimental evidence suggests that ORF1p forms a homotrimer that then coats the LINE-1 mRNA in a multitude of copies. [61]

**Fig. 6:**
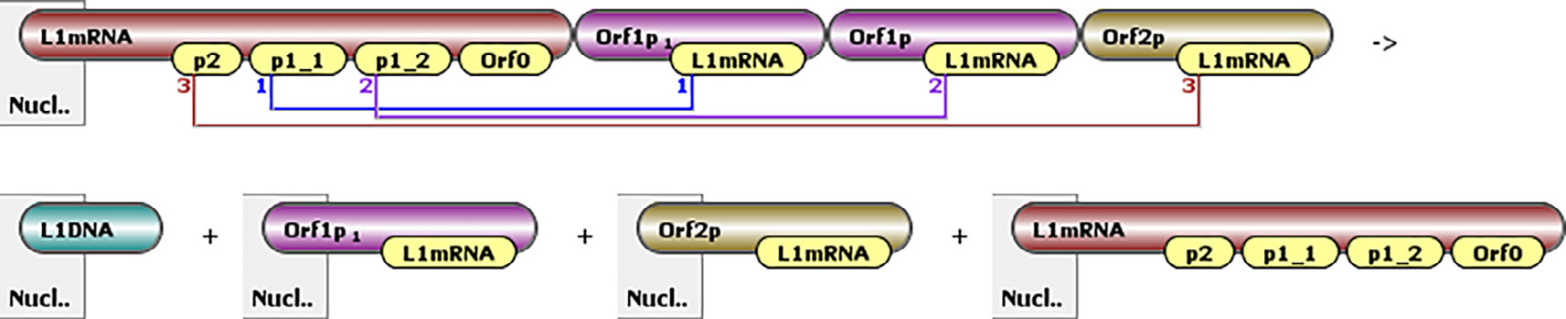
Example visualization of a reaction rule where reverse transcription of LINE-1mRNA that was complexed with an ORF2p and 2 ORF1p proteins catalyzes a new copy of LINE-1 DNA. After the reaction, the constituents completely disassociate.

BioNetGen was used to calculate all the permutations of reactions that may be possible with multistate molecules based on the defined reaction rules. An example of a reaction rule definition from the model is depicted in Fig. 6, where LINE-1mRNA is complexed with an ORF2p and two ORF1p proteins but is NOT complexed with an ORF0 mRNA. The complex undergoes an irreversible reaction to catalyze a new LINE-1 DNA copy after which the RNP completely disassociates.

Given that ORF1p and ORF2p do not localize in the nucleus experimentally, our model posits that these two proteins will complex with LINE-1 mRNA only in the cytosol, as opposed to complexing with newly transcribed LINE-1 mRNA in the nucleus after finishing a round of reverse transcription. Reactions involving the degradation of ORF1p and ORF2p are assumed to take place in the cytoplasm or in stress granules (as passengers on LINE-1mRNA) as these proteins are generally not found in the nucleus. Similarly, LINE-1mRNA degradation within the model occurs in the cytoplasm through a general background RNase reaction or inside the stress granules. This assumption was made as the reverse transcriptase in ORF2p does not have RNase H activity, although there is evidence that endogenous RNase H2 plays a role in nuclear LINE-1 mRNA degradation [25]. The MOV10_Zap protein may interact with the LINE-1mRNA that has occupied ORF1p and ORF2p sites, as it complexes via the ORF0 mRNA site.

## 9 Results using the full model

**Fig. 7:**
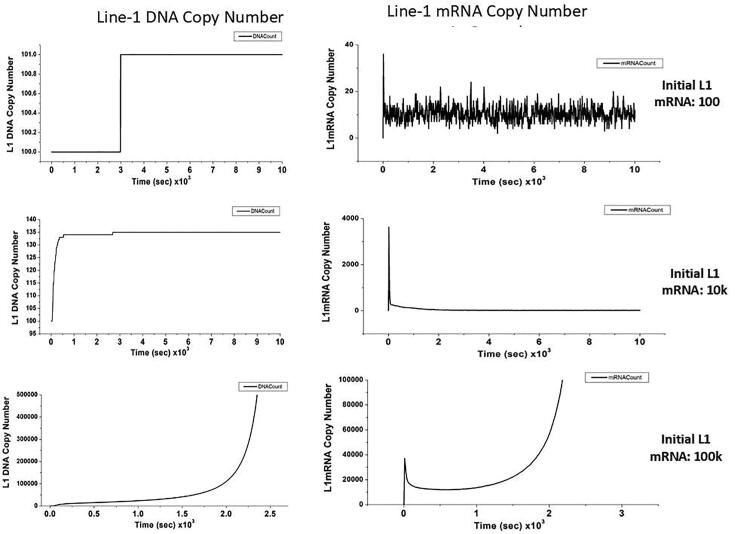
Evolution of LINE-1 DNA and cytoplasmic mRNA for different initial numbers of nuclear LINE-1 mRNA. The various numbers of initial nuclear mRNA model various degrees of LINE-1 DNA activation after acute exposure to carcinogens.

The default initial conditions for many of the simulations are shown in Table 1, with all unlisted species set to zero molecules. Typical simulations were run for 10,000 s (2.7 h) and solutions result in the time dependence of all species and reaction fluxes. In an effort to simulate the acute exposure of cultured human (HBEC) cells to benzo(a)pyrene (B(a)P), a transcriptional activator of LINE-1, we performed a parameter sweep that varied the copy number of initial LINE-1 mRNA from 0 to 100,000 in decade steps. Here, the assumption was that an acute exposure to various doses of the carcinogen result in transient expression of LINE-1 mRNA and subsequent increases in copy number. The simulation indicated that there is a definite threshold where the copy number of LINE-1 DNA transitions from no change, then to a modest increase and eventually, at 100 k copies of mRNA, the LINE-1 DNA copy number falls into a positive feedback loop, overwhelming the cellular defenses, resulting in an exponential increase. Fig. 7 shows the time evolution of LINE-1 DNA and cytoplasmic mRNA count for simulations having 100, 10,000 and 100,000 initial copies of LINE-1 mRNA in the nucleus. In the top row of the figure, one can see that the number of DNA copies increased by only one when the initial number of nuclear LINE-1 mRNA is only 100. In the top right, the LINE-1 mRNA in the cytosol first rises to about 39 copies but quickly falls to a stable basal level of 5–20 copies. In the center row, corresponding 10,000 initial mRNA copies, the LINE-1 DNA copy number actually rises to 135 and then stabilizes by the end of the simulation. Similarly, the LINE-1 mRNA in the cytosol first rapidly rises and once again, drops to a basal level of 15–20 copies by the end of the simulation. Finally, in the bottom row with an initial nuclear LINE-1 mRNA count of 100,000, the growth of both DNA and RNA becomes exponential causing the simulation to fault out before reaching the 10,000 s end point. It is interesting to note that only in the last case of 100,000 LINE-1 mRNA copies does there seem to be any role for RISC degradation or sequestration.

**Fig. 8:**
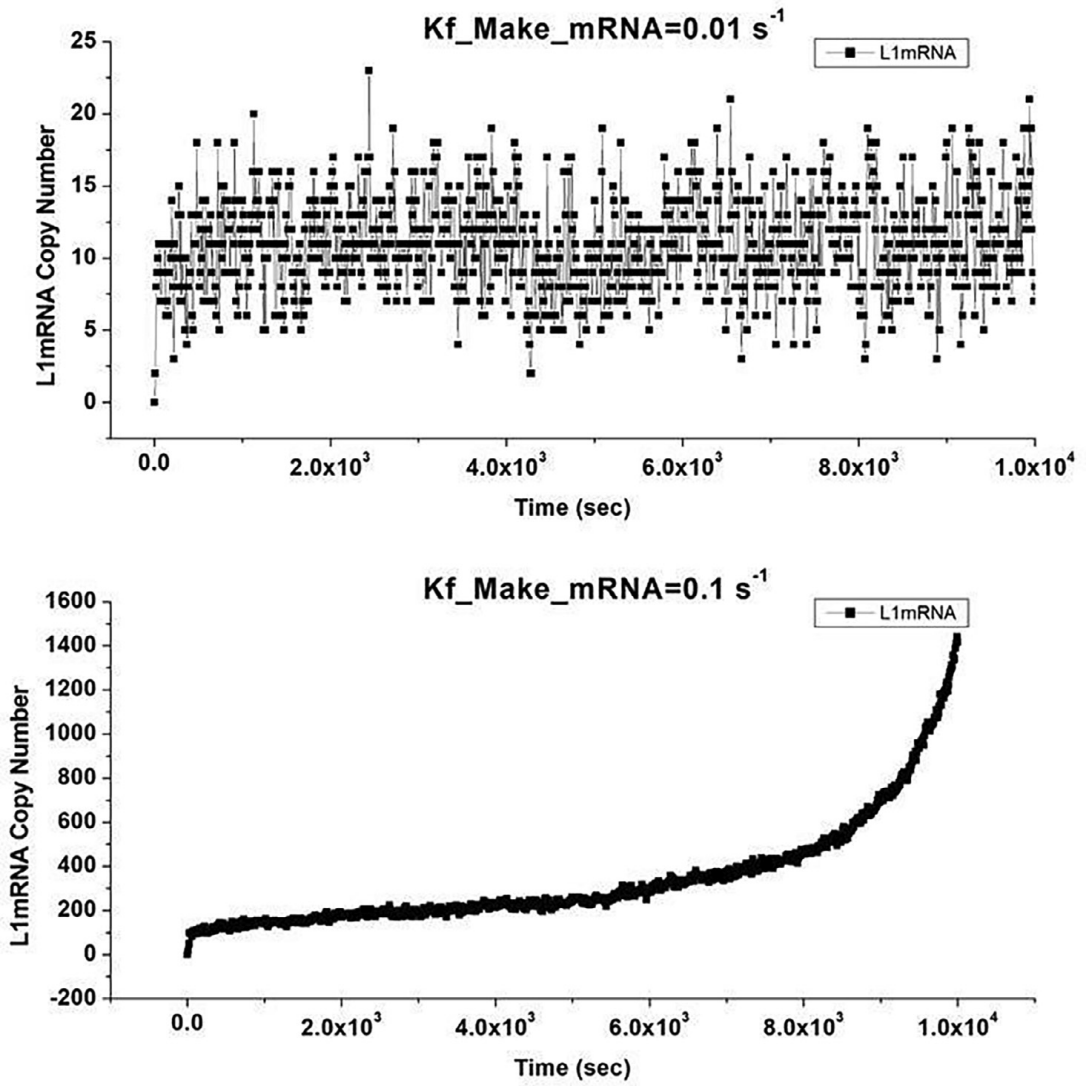
The influence of mRNA transcription rate on LINE-1 mRNA copy number. In the top graph, the amount of mRNA in the cytoplasm remains roughly constant at a mRNA synthesis rate of 0.01 s^−1^ but begins to grow exponentially when the rate reaches a threshold of 0.1 s^−1^ as shown in the lower graph.

In a second set of simulations the role of the transcription rate of LINE-1 mRNA was explored as a proxy for epigenetic dysregulation of LINE-1 due to carcinogen exposure. The constant, Kf_Make_L1mRNA, was varied from 0.0001 s^−1^ to 1.0 s^−1^ in decade steps and the model run for 10,000 s. Again, the model showed a distinct threshold for the growth in LINE-1 DNA copy number between 0.01 s^−1^ and 0.1 s^−1^ and similarly the copy number of LINE-1 mRNA shows threshold behavior as illustrated in Fig. 8. As in the previous case where the initial number of LINE-1 mRNA was varied, the role of RNAi and sequestration is minimal compared to general RNAse activity in regulating LINE-1 mRNA. Indeed, in all cases where the LINE-1 mRNA creation rate was varied, RNAi showed no LINE-1 mRNA degradation events even after the rate of LINE-1 DNA creation began to feed forward exponentially. In contrast, the stress granule and multivesicular body localization pathways began to be utilized at the three highest values of the LINE-1 mRNA creation rates (i.e., 0.01 s^−1^, 0.1 s^−1^ and 1.0 s^−1^). Given that the reported value of the transcription rate of ORF0 is approximately 1/10 the transcription rates of the LINE-1 mRNA for ORF1 and ORF2 [14], two rates of ORF0 creation were explored, 0.001 s^−1^ and 0.1 s^−1^. These values reflect the ORF1 and ORF2 creation rate extrema of 0.01 s^−1^ and 1.0 s^−1^ mentioned above. In both cases we did not observe RISC degradation events over the model period of 10,000 s.

One element of the model that may be of concern when LINE-1 mRNA copy number begins to get large, is the finite number of molecules such as Exportin, Mov10, Dicer, and RISC with initial copy numbers of 1000. The model was thus also run with constant copy numbers such that when a species, such as Exportin left the nucleus, a new copy would be replenished to keep the copy number in the nucleus constant. The model retained the same qualitative behavior and exhibited the same threshold values for exponential growth as a function of the LINE-1 mRNA creation rate and were nearly identical when we examined various initial values for the number of LINE-1 mRNA copies.

## 10 Conclusions

Previous experiments have demonstrated that the time course of LINE-1 activation upon exposure to B(a)P in HBEC lines peaks at 12 h and returns to baseline after 48 h [37]. The present model shows similar behavior, where the sweep of initial LINE-1 mRNA count (Fig. 7) shows that typical cellular defenses are capable of preventing the uncontrolled proliferation of LINE-1 up to a certain critical load of initial mRNA. Unlike the findings derived from experimental data, the kinetics of the model are faster and show that the mRNA count drops within the first 100 s to a basal level for all but the largest values of mRNA. This is likely due to our qualitative estimate of many model parameters such as LINE-1 transcription and translation rates, nuclear transport kinetics for species, LINE-1 ORF protein-RNA binding kinetics and RNase kinetics. Additionally, damage to the epigenetic regulators is modeled as a one-time increase in the number of LINE-1 mRNA copies where it would be more accurate to model these effects with an empirically informed, time varying LINE-1 mRNA transcription rate. The model also does not include LINE-1 catalyzed proliferation of the far more numerous, non-autonomous retroelements or Short Interspersed Nuclear Elements (SINEs). RNA from SINEs may act as a sink for ORF2p and other components of cellular post transcriptional suppression, thus slowing the dynamics of LINE-1.

In the case of simulations that explored the creation rate of LINE-1 mRNA, the model once again exhibited threshold behavior above which the feed-forward loop of LINE-1 results in exponential growth of its mRNA and DNA copy number. Clearly, the uncontrolled growth of these factors is not physical and would be limited by the availability of cellular resources such as nucleotides, amino acids, and tRNA. The model would be improved by including other sources of siRNA such as non-autonomous copies of LINE-1 with functional antisense ORF0 or other endogenous siRNAs. [12] Further, the utility of this model would be greatly enhanced if the epigenetic mechanisms controlling LINE-1 expression were developed to reflect histone modifications, DNA methylation and the role of the PIWI system.

One interesting consequence that is evident from the model architecture is that once the LINE-1 mRNA is bound by ORF1p or ORF2p, translation stops as ribosomes are no longer able to bind the mRNA. This finding calls for further evaluation in light of studies by Alisch et al. [62] implicating an unconventional translation/re-initiation pathway for L1 translation, wherein multiple ORF1p molecules are translated from and coat a single L1 mRNA molecule. In their study, however, inhibition of translocation of scanning ribosomes was shown to reduce ORF2p synthesis, a finding consistent with our model. In discussing this work, Dmitriev et al. [63] noted that the start of translation of ORF2p was selected by an unconventional mechanism of reinitiation that did not involve an internal ribosomal entry site, with procession of the ribosomal complex interrupted by the presence of ORF1p or ORF2p on Line-1 mRNA. Additionally, our model suggests that ORF1p and ORF2p may inhibit RNAi by binding to their respective sites when LINE-1 mRNA is complexed with ORF0 mRNA to prevent Dicer loading. This protective role for ORF1p against other degradation pathways has been confirmed experimentally. [61] The model further showed that the RNAi pathway, using empirically derived reaction constants, and the antisense ORF0 RNA, did not play a significant role in decreasing the rate of retrotransposition. Thus, the model does not explain empirical observations showing that transcription of ORF0 mRNA increases retrotransposition. Given that the ORF0 protein is not addressed in the model, future work should include definitive roles for ORF0 in the life cycle of LINE-1. Additionally, the number of RISC and DICER molecules is set to a constant, while the relevant molecules would likely be subject to positive regulation as the pathway is utilized. There may also be siRNAs and piRNAs that interact with LINE-1 mRNA to yield processing in RISC and the model should be refined in the future to reflect these factors [14]. Furthermore, experimentally there is a complex relationship between the 5′ antisense RNA, L1-ORF1p, Argonaut proteins and siRNA-mediated regulation of LINE-1 [64]. In the same study, the authors demonstrated that not only does the antisense RNA reduce sense 5′ UTR expression, but that L1-ORF1p directly binds to Argonaut proteins- even without RNA as a mediator. Future models should include these interactions to form a more complete picture of LINE-1 dynamics.

Unlike the RNAi pathway, those involving sequestration to multi-vesicular bodies and stress granules were utilized when LINE-1 mRNA creation rates were 0.01 s^−1^ and above. It seems that these metabolically costly pathways only become important as the RNase within the cytosol becomes overwhelmed. The current instance of the model lumps together a number of independent pathways related to RNP, RNA and protein sequestration into two broad categories: multivesicular bodies and stress granules using only a single protein as a place-holder for a far more complex group of processes. As such, this system should be represented with far more fidelity in future work. As noted earlier, LINE-1 is often activated in cancer cells, although the exact relationship between cancer and retrotransposon activity has not been fully elucidated. This is particularly relevant in trying to elucidate the role of LINE-1 laden exosome formation in cancer and studies of the role of processing bodies in the formation of LINE-1 RNPs. As genomic databases continue to expand, these data can be used to refine the model by exploring critical genomic and epigenomic interactions between cancer genes and LINE-1. A number of open questions were illuminated in our study. For example, what happens to the components of the LINE-1 RNP after reverse transcription in the nucleus? Particularly, if ORF2p does not degrade LINE-1 RNA upon reverse transcription [9], [59], why is it that new RNP’s do not form in the nucleus? Experimentally, this does not seem to occur given the lack of ORF1p and ORF2p accumulation in the nucleus and there is speculation that RNPs may only form in processing bodies [42]. Given that the formation of LINE-1 mRNA complexes with ORF1p and ORF2p is likely to block RNAi and sequestration into stress granules, it might be interesting to experimentally explore delivery of short RNA sequences that are homologous to LINE-1mRNA to block the interactions. Finally, some studies suggest that LINE-1 proliferation is closely tied to the cell cycle and dissolution of the nuclear membrane [65], thus future models should explore this relationship.

## CRediT authorship contribution statement

**Michael David Martin:** Conceptualization, Methodology, Writing - original draft, Writing - review & editing, Visualization. **David N. Brown:** Conceptualization, Writing - review & editing, Supervision, Project administration. **Kenneth S. Ramos:** Conceptualization, Writing - review & editing, Supervision, Project administration, Funding acquisition.

## Declaration of Competing Interest

The authors declare that they have no known competing financial interests or personal relationships that could have appeared to influence the work reported in this paper.
